# Frailty assessment in primary health care and its association with unplanned secondary care use: a rapid review

**DOI:** 10.3399/bjgpopen18X101325

**Published:** 2018-04-10

**Authors:** Ben R Davies, Helen Baxter, James Rooney, Phillip Simons, Ann Sephton, Sarah Purdy, Alyson L Huntley

**Affiliations:** 1 Senior Research Associate, Centre for Academic Primary Care, Population Health Sciences, Bristol Medical School, University of Bristol, Bristol, UK; 2 Senior Research Associate, Centre for Academic Primary Care, Population Health Sciences, Bristol Medical School, University of Bristol, Bristol, UK; 3 Senior Project Manager, NHS South, Central and West Commissioning Support Unit, Bristol, UK; 4 Clinical Evidence Fellow, NHS South Gloucestershire Clinical Commissioning Group, Warmley, UK; 5 Deputy Clinical Chair and Lead for Emergency and Urgent Care and Community Health Services, NHS South Gloucestershire Clinical Commissioning Group, Warmley, UK; 6 Professor of Primary Health Care, Centre for Academic Primary Care, Population Health Sciences, Bristol Medical School, University of Bristol, Bristol, UK; 7 Research Fellow, Centre for Academic Primary Care, Population Health Sciences, Bristol Medical School, University of Bristol, Bristol, UK

**Keywords:** frailty assessment, geriatric assessment, primary heath care, hospital admission

## Abstract

**Background:**

The growing frail, older population is increasing pressure on hospital services. This is directing the attention of clinical commissioning groups towards more comprehensive approaches to managing frailty in the primary healthcare environment.

**Aim:**

To review the literature on whether assessment of frailty in primary health care leads to a reduction in unplanned secondary care use.

**Design & setting:**

A rapid review involving a systematic search of Medline and Medline In-Process.

**Method:**

Relevant data were extracted following the iterative screening of titles, abstracts, and full texts to identify studies in the primary or community healthcare setting which assessed the effect of frailty on unplanned secondary care use between January 2005–June 2016.

**Results:**

The review included 11 primary studies: nine observational studies; one randomised controlled trial (RCT); and one non-randomised controlled trial (nRCT). Eight out of nine observational studies reported a positive association between frailty and secondary care utilisation. The RCT and nRCT reported conflicting findings.

**Conclusion:**

Older people identified as frail in a primary healthcare setting were more likely to be admitted to hospital. Based on the limited and equivocal trial evidence, it is not possible to draw firm conclusions regarding appropriate tools for the identification and management of frail older people at risk of hospital admission.

## How this fits in

A growth in the number of frail, older individuals is directing attention towards the use of a comprehensive geriatric assessment (CGA) in primary health care. This review explored the assessment of frailty in the primary healthcare setting and its association with unplanned secondary care admissions, for which there are currently no published reviews. Eight of nine observational studies reported a positive association between frailty and secondary care use. One RCT and one nRCT presented equivocal findings. More evidence is needed on frailty assessment and the use of a CGA in primary health care, as well as the acceptability of such tools to the primary healthcare workforce.

## Introduction

Frailty is a distinct health state associated with the ageing process;^1^ it is characterised by a loss of biological reserves throughout multiple organ systems and susceptibility to physiological decompensation after a potentially minor health event.^2^ Although a definitive operational definition of frailty is yet to be agreed on, two conceptual models dominate the field: The Frailty Index (FI)^3^ and the Frailty Phenotype (FP).^4^ The FI identifies frailty as a state, defined as an accumulation of deficits over time. The FI’s deficits comprise an adaptable range of conditions and diseases, from physical to psychosocial.^3^ The FP distinguishes frailty as a syndrome identified by a pre-defined set of five criteria: involuntary weight loss; exhaustion; slow gait speed; poor handgrip strength; and sedentary behaviour.^4^

There is global concern that existing healthcare services cannot meet the demand of an increasing frail population.^5^ However, frailty is not an inevitable part of ageing and the condition can be improved through appropriate management.^1^ One suggested approach to tackling the challenges of an ageing population is primary prevention.^5^ Conceptual models such as the FP and FI have led to the development of several frailty assessment instruments, which provide the opportunity to develop interventions against such age-related conditions. One such approach to manage frailty is the CGA, the purpose of which is to conduct a holistic, interdisciplinary, and multidimensional frailty assessment,^1^ and subsequently develop a management plan, comprising treatment and follow-up, linking medical and social care.^6^

The British Geriatric Society (BGS) suggests that healthcare professionals assess frailty during routine primary healthcare encounters and then refer to a geriatrics team to perform a CGA.^1^ There is evidence that the use of a CGA to guide treatment significantly improves the chances of a patient being alive and in their own home 12 months after an emergency hospital admission,^7^ and this is of increasing interest to primary healthcare policy makers. A shortened form of the CGA has been developed for use in primary health care with the objective of identifying frail individuals most at risk of requiring secondary care admission.^8^ Despite there being some evidence for the effectiveness of conducting frailty assessments within primary health care and its role in reducing hospital admissions, there is no evidence to date supporting the specific use of a shortened CGA in that setting, as far as the authors are aware.

Considering this, a rapid review of the literature was conducted to identify if assessment of frailty and/or management of frailty, including CGA, in PHC lead to reductions in unplanned secondary care use.

## Method

The literature search followed standard methodology informed by the latest Cochrane systematic review guidance.^9^ The authors searched Medline and Medline In-Process from 1 January 2005–8 June 2016, and identified 984 entries. The search structure is available from the authors on request.

### Eligibility criteria

The search strategy sought studies meeting the following criteria:

- Population/intervention: the population included frail adults receiving an intervention involving any type of frailty assessment conducted by primary or community healthcare professionals.
- Control: any type of control group.
- Outcome: any measurement assessing the effect of frailty on unplanned secondary care use.
- Design: quantitative and qualitative studies.

Non-English language studies were considered if they had an English abstract on which to assess their eligibility.

References were managed using EndNote software and screened by two authors using the above eligibility criteria. Abstracts were initially screened and then full papers of potential studies were screened to produce the final inclusion list. Any disagreements at either stage were resolved using a third reviewer.

Data were extracted into a custom-designed table to capture all relevant information. The Cochrane Collaboration’s tool for assessing risk of bias was used to assess risk of bias for trials identified in the rapid review.^10^

## Results

The review identified 11 primary studies: nine observational studies; one RCT; and one nRCT. More study detail is provided in a brief summary of the observational and trial evidence (Table 1). Seven studies were conducted in Western Europe, including the Republic of Ireland and Northern Ireland,^11^ Spain,^12^ the Netherlands,^13,14^ Switzerland,^15^ Portugal,^16^ and the UK.^2^ Two studies were conducted in Australia,^17,18^ one in Singapore,^19^ and one in the US.^20^ Risk of bias was generally low for both RCTs and high for the nRCT, due to its lack of randomisation. No relevant qualitative studies were identified.

**Table 1.** Summary of evidence for included studies

	Study design and aims	Eligibility criteria or population	Frailty assessment	Secondary care use
**Observational evidence** **Retrospective studies**				
Clegg 2016,^2^ UK	**Design**: 5-year retrospective cohort (2008–2015). **Aim**: Development and validation of an electronic frailty index (eFI) using primary care electronic health record data from the THIN database.	Individuals aged 65–95 years registered to a Research One or THIN database practice. **Development cohort** (*n* = 207 814) **Internal validation cohort** (*n* = 207 720) **External validation cohort** (*n* = 516 007)	An FI was created using the cumulative deficit model in a randomly split sample of the Research One database.	**External validation cohort**Hazard ratios (with 95% CI) for risk of unplanned hospital admissions for each degree of frailty: **Year 1** Mild 2.03 (1.96 to 2.10) Moderate 3.50 (3.38 to 3.63) Severe 5.58 (5.34 to 5.84) **Year 3** Mild 1.89 (1.85 to 1.93) Moderate 3.03 (2.96 to 3.11) Severe 4.66 (4.51 to 4.80)
Donate-Martinez 2014,^12^ Spain	**Design:** 12-month retrospective (2008–2009). **Aim:** To determine the viability of the implementation of two screening tools — namely, the Probability of Repeated Admission (Pra) and the Community Assessment Risk Screen (CARS) — to detect patients at risk of hospital readmission.	Community-dwelling patients in Spain aged ≥65 years. (*n* = 500)	Data related to the variables that comprise both Pra and CARS were collected with a reference date of December 2008. Pra was categorised as high, medium, and low risk of admission. CARs was categorised as high and low risk.	**Admissions, mean (SD):** **Pra** *P*≤0.001 High 0.47 (0.86) Medium 0.25 (0.61) Low 0.12 (0.45) **CARS** *P*≤0.001 High 0.36 (0.76) Low 0.11 (0.40) **Length of stay (days), mean (SD):** **Pra** *P≤*0.001 High 2.29 (7.72) Medium 0.98 (3.00) Low 0.43 (2.08) **CARS** *P* ≤0.001 High 1.76 (5.28) Low 0.44 (2.01) **ROC curve analysis, AUC**: Pra: 0.67 CARS: 0.69
**Cross-sectional studies**				
Dent 2016,^17^ Australia	**Design:** Secondary cross-sectional. **Aim:** To investigate specific health service provision among frail older people in the rural community of Port Lincoln, south Australia.	Participants aged ≥65 years who completed the LINKIN health study health census September–November 2010. (*n* = 1796)	An FI of cumulative deficits was used to classify frailty. Frailty guidelines were used to construct an FI of 23 variables falling into three categories: comorbidities, functional measures, and quality of life. Frailty was dichotomised into frail and non-frail. Participants with ≥6 accumulated deficits were considered to be frail. Participants with one or more missing FI variables were excluded from final dataset (*n* = 1501 for final dataset).	Hospital admission as an inpatient over previous 12 months, by frailty category (*n* = 1490). ***n* (%):** **Non-frail** 73 (5) **Frail 55** (15) OR = 2.39 (95% CI = 1.74 to 3.29) *P*<0.001
Ng 2014,^19^ Singapore	**Design:** Cross-sectional with 2-year validation follow-up. **Aim**: Development of a simple frailty risk index (FRI), and evaluation for use in primary care on an external validation cohort of community-living older persons.	Adults aged ≥55 years in the Singapore Longitudinal Ageing Studies. **Development cohort** *n* = 1685 **Validation cohort** *n* = 2478	The FRI was developed by identifying 13 salient and independent multisystem risk factors for the 5-criteria frailty phenotype used in the Cardiovascular Health Study (CHS): weakness, slowness, low physical activity, weight loss, and exhaustion. A risk score was assigned to each risk factor and the FRI was derived from summating risk scores associated with each risk factor.	**Validation cohort** Association of frailty defined by the FRI (as continuous variable) with self-reported hospitalisations: OR = 1.14 (95% CI = 1.05 to 1.24), *P* = 0.002 ROC curve analysis, AUC: 0.63
Rochat 2010,^18^ Australia	**Design:** Cross-sectional. **Aim:** To describe the relationship between frailty and use of several health and community services in community-dwelling older men.	Men aged ≥70 years in the Concord Health and Ageing in Men Project.	Frailty was assessed using a modified version of the CHS criteria: Weight loss or shrinking, weakness, exhaustion, slowness, and low activity. Robust was categorised as meeting 0 criteria; pre-frail as ≤2 criteria; and frail as ≥3 criteria.	Participants admitted for ≥1 night in hospital during previous 12 months, *n* (%): **Robust** 152 (18.2), AOR 1.00 (reference) **Pre-frail** 174 (25.7), AOR 1.34 (95% CI = 1.03 to 1.74) **Frail** 81 (51.6), AOR 3.29 (95% CI = 2.18 to 4.96)
McGee 2008,^11^ Ireland	**Design:** Cross-sectional. **Aim:** To assess if those categorised as vulnerable by the Vulnerable Elders Survey (VES) were likely to use health services more frequently than others.	Randomly selected community-dwelling individuals aged ≥65 years living at a private residential addresses and able to participate in a research interview. (*n* = 2033)	The VES is a 13-item questionnaire developed from studying >6000 community-dwelling Medicare beneficiaries aged ≥65 years.	Health service use in previous 12 months (*n* = 2033), by VES score: High VES score (32.1% of sample) versus low VES score (67.9% of sample) **Emergency department visits**: 17% versus 8% *P*<0.001 **Scheduled hospital inpatient stay**: 21% versus 12%, *P*<0.001
**Longitudinal studies**				
van Kempen 2015,^13^ Netherlands	Des**i**gn: 1-year longitudinal cohort (2010–2011). **Aim:** To determine the predictive value of EASY-Care Two Step Older Persons Screening (EASY-Care TOS) for negative health outcomes within 12 months from assessment.	Patients aged ≥70 years from participating GP practices. (*n* = 520)	Participants were assessed with the complete EASY-Care TOS procedure. All subsequent assessment steps were finished, irrespective of the outcome (usually a two-step process), during routine primary care visits. Patients were assigned as frail or not-frail.	Hospital admission during previous 12 months for participants classified as frail (*n* = 195) or not frail (*n* = 325), *n* (%): **Frail** 39 (22.0) **Not frail** 41 (12.9) Absolute difference = 9.1 (95% CI = 2.0 to 16.2), *P* = 0.01.
Sha 2005,^20^ US	**Design:** Cross-sectional. **Aim:** To describe the patterns of physical symptoms in older adults and to examine the validity of symptoms in predicting hospitalisation and mortality.	Individuals aged ≥60 years completing a screening for self-reported symptoms during a routine primary care visit. (*n* = 3498)	Self-reported symptoms were collected using an abbreviated primary healthcare evaluation of mental disorders screening instrument (PRIME-MD).	Hospitalisations in the year following screening according to medical records by number of reported symptoms, *n* (%): **0–2 symptoms** 171 (16.2) OR = 1.0 (reference) **3–4 symptoms** 191 (20.9) OR = 1.2 (95% CI = 0.9 to 1.5) **5–7 symptoms** 218 (22.3) OR = 1.2 (95% CI = 0.9 to 1.5) **8–12 symptoms** 154 (27.9) OR = 1.4 (95% CI = 1.0 to1.9)^a^
Coelho 2014,^16^ Portugal	**Design:** 10-month longitudinal. **Aim:** To compare how different frailty measures predict short-term adverse outcomes, namely Frailty Phenotype (FP), Groningen Frailty Indicator (GFI), and Tilburg Frailty Indicator (TFI).	Individuals aged ≥60 years based in the community (*n* = 252)	Part A of TFI was used to assess life-course determinants of frailty and comorbidity, while FP, GFI, and part B of TFI were used to measure frailty.	Hospitalisations in the previous 12 months χ^2^: **FP** *P* = 0.29^b^ **Non-frail, *n* (%)** Yes: 6 (54.5) No: 61 (72.6) **Frail, *n* (%)** Yes: 5 (45.5) No: 23 (27.4) **GFI** *P* = 0.08 **Non-frail, *n* (%)** Yes: 3 (27.3) No: 46 (54.8) **Frail, *n* (%)** Yes: 8 (72.7) No: 38 (45.2) **TFI** *P* = 0.09 **Non-frail, *n* (%)** Yes: 3 (27.3) No: 46 (54.8) **Frail, *n* (%)** Yes: 8 (72.7) No: 38 (45.2)
**RCT and nRCT evidence**				
	**Study design and aims**	**Eligibility criteria or population**	**Frailty assessment or group assignment**	**Trial outcome** **(secondary care use)**
Ruikes 2016,^14^ Netherlands	**Design:** Two-arm, 12-month nRCT (September 2011–September 2012). **Aim:** To evaluate the effectiveness of a GP–led extensive, multicomponent programme for the prevention of functional decline. **Risk of bias**: High.	GP practices with sufficient numbers of patients aged ≥70 years and adequate facilities to enable programme implementation. Exclusion criteria: Admission to a residential or nursing home and/or critical or terminal illness (*n* = 536).	Six intervention practices were informed about the programme and six control practices were not (usual care). After Easy-Care TOS assessment, GP and practice nurse or research assistant made a final decision on the presence of frailty. Intervention: The Care Well primary care programme, consisting of four key elements: multidisciplinary team meetings, proactive care planning, case management, and medication review.	Data collected at baseline and 12 months through a home visit by either a trained nurse (in the intervention arm) or a research assistant (in the control arm). Hospital admissions during follow-up, *n* (%): **Intervention**: 52 (18.1) **Control:**57 (22.9) OR **=** 0.74 (95% CI = 0.48 to 1.14), *P* = 0.17.
Imhof 2012,^15^ Switzerland	**Design:** RCT. **Aim:** To evaluate the effects of a 9-month advanced practice nurse (APN) in-home health consultation programme (HCP) on quality of life and health. **Risk of bias**: Low.	Inclusion criteria: German-speaking, community-dwelling individuals aged ≥80 years, cognitively able to understand study and consent. Exclusion criteria: End of life, with a major psychiatric diagnosis, or severe cognitive impairment. Intervention: *n* = 231	Participants were randomly assigned to the intervention or control group following two baseline assessment visits with an APN, which included a standardised comprehensive geriatric assessment (CGA). All participants received health care as usual. Intervention: In addition to usual care, a complementary 9-month in-home HCP was delivered by one of four APNs. The HCP comprises a CGA and consultations that identify and consider the health problems and concerns of the participants. The intervention included four home visits (mean length 46 ± 6 minutes) after 4, 12, 24, and 36 weeks, and three telephone calls (mean length 17 ± 4 minutes) after 8, 18, and 30 weeks. Total intervention time per participant averaged 4 hours.	Outcomes were collected at 3, 6 and 9 months. With regard to hospitalisations, multilevel analysis showed no evidence of effect for intervention (*P* = 0.86), time course (*P* = 0.33), or interaction (*P* = 0.24). However, hospitalisations (number of 3-month periods with a planned or unplanned hospital admission or emergency department visit) were lower in the intervention group, *n* (%): **Intervention versus control** 47 (23) versus 68 (33) Relative risk = 0.70 Numbers needed to treat = 10.0, *P* = 0.03.

^a^P<0.05. ^b^Fisher’s exact test. AUC = area under curve. AOR = adjusted odds ratio. nRCT = non-randomised controlled trial. OR = odds ratio. RCT = randomised controlled trial. ROC = receiver operating characteristic.

Of the nine observational studies identified, eight reported a positive association between frailty and secondary care utilisation. The review identified only one RCT and one nRCT; in these trials frailty assessment preceded an intervention.^11,12^ Both trials, however, were considered relevant given the application of the frailty assessment to the care received by intervention participants.

### Retrospective studies

The electronic health record data of individuals aged 65–95 years from the UK was used to develop and validate the electronic Frailty Index (eFI).^2^ The eFI was externally validated in 516 007 primary healthcare patients (mean age 75 ± 7.3 years) over a 3-year period. At the 3-year time point, hazard ratios demonstrated that patients with ‘severe’ frailty were at 4.66 (95% confidence interval [CI] = 4.51 to 4.80) times greater risk of an unplanned secondary care admission than those identified as ‘fit’. The eFI predicted secondary care utilisation with fair predictive validity at year 1 (cStatistic 0.71), although the calibration estimate (variance explained by eFI) for the hospital admissions outcome was low. The eFI algorithm was incorporated into the System One GP health record system for feasibility testing in 2014, and in 2016 was introduced via the EMISWEB and Vision clinical systems.^21^

A Community Assessment Risk Screen (CARS) and the Probability of Repeated Admission (Pra) tool was used to detect hospital admission risk of 500 community-dwelling patients in Spain aged ≥65 years.^12^ Those classified at higher risk of admission by both the CARS and Pra tools reported more per-patient hospital admissions (*P≤*0.001) and greater lengths of hospital stay (*P≤*0.001) in the subsequent 12 months. However, poor predictive values (area under the curve [AUC] and positive predictive values [PPV]) suggest neither tool efficiently identifies risk of secondary care utilisation.

### Cross-sectional studies

An Australian study utilised the data of 1501 individuals aged ≥65 years to identify frailty by applying the FI model.^17^ Participants classed as frail by the FI were 2.39 (95% CI = 1.74 to 3.29) times more likely to be admitted to hospital compared to non-frail participants during the previous 12 months (*n* = 1490 for participants with hospital admissions data).

The Cardiovascular Health Study (CHS) criteria (weight loss or shrinking, weakness, exhaustion, slowness, and low activity) were used to identify frailty in 1674 men aged ≥70 years, living in Sydney, Australia.^18^ Compared to ‘robust’ men (0 frailty criteria), ‘frail’ men (≥3 frailty criteria) were 3.29 (95% CI = 2.18 to 4.96) times more likely to spend ≥1 night admitted to hospital during the previous 12 months.

Data from the Singapore Longitudinal Ageing Study was used for the development of a primary healthcare clinical frailty risk indicator (FRI) in 1685 patients (mean age 67 ± 8 years).^19^ The development study evaluated how frailty risk factors predict frailty, as defined by five criteria validated in the CHS.^4^ In the validation cohort (*n* = 2478), participants were 1.14 (95% CI = 1.05 to 1.24; *P* = 0.002) times more likely to be admitted to hospital during the 2-year follow-up period per unit increase in FRI score. However, the predictive validity of the FRI with regards secondary care utilisation was poor (AUC = 0.63).

A further study investigated the association between responses of the Vulnerable Elders Survey (VES) and secondary care utilisation in 2033 patients aged ≥65 years from Ireland.^11^ Over the previous 12 months, hospital inpatient stays had been reported more frequently (21% versus 12%, *P≤*0.001), as were emergency department visits, in those classed by the VES as ‘high’ compared to ‘low’ vulnerability.

### Longitudinal studies

In a longitudinal study from the Netherlands, 520 patients (mean age 77 ± 5 years) were assessed for frailty by their GP at baseline, using the EASY-Care Two step Older persons Screening (EASY-Care TOS) tool.^13^ During the 12-month follow-up, those classed as ‘frail’ reported a greater proportion of hospital admissions than their ‘non-frail’ counterparts (39 [22%] versus 41 [12.9%], *P* = 0.01).

The PRIME-MD (primary healthcare evaluation of mental disorders) screening instrument was employed in a study from the US exploring the validity of physical symptoms identified during routine primary healthcare visits for predicting hospitalisations in 3498 adults (mean age 69 ± 7 years).^20^ Compared to those with 0–2 symptoms, participants with 8–12 symptoms were 1.4 times more likely to be admitted to secondary care, (95% CI = 1.0 to 1.9, *P*≤0.05).

However, in a study of 252 community-based participants aged ≥60 years there was no evidenced association between frailty and secondary care utilisation.^16^ The authors compared three frailty measures: the FP, Groningen Frailty Indicator, and Tilburg Frailty Indicator for the prediction of secondary care utilisation over the previous 12 months. Given the small sample size at follow-up (*n* = 95), the authors highlight their study’s lack of statistical power.

### Controlled trials

An nRCT of The Care Well primary healthcare programme based in the Netherlands found no evidence of effect in moderating hospital admission rates in the intervention compared to control group.^14^ This 12-month intervention was conducted in 536 participants (intervention *n* = 287, control *n* =249) aged ≥70 years who were identified as frail using the Easy-Care TOS tool. The trial was non-randomised and lasted 12 months, which may have been insufficient for establishing effective multidisciplinary collaborations.

In Switzerland, an in-home health consultation programme was delivered by advanced practice nurses (APNs) to 461 (intervention *n* = 231, control *n* = 230) German-speaking, community-dwelling individuals aged ≥80 years, and was evaluated over 9 months.^15^ Multilevel analysis showed no evidence of effect, although group comparisons showed overall secondary care admission rates were lower in the intervention compared to control group (47 [23%] versus 68 [33%], *P* = 0.03, relative risk ratio = 0.70). The lack of change in other healthcare services suggested the intervention did not replace existing services but was complementary to them. The study employed un-blinded data collection and self-reported measures over 3-month periods, and thus reporting bias and misreporting is a possibility.

### Reviews

An International Academy of Nutrition and Aging taskforce reviewed the evidence for gait speed assessed at usual pace to identify risk of adverse outcomes in community-dwelling older people.^22^ Twenty-seven articles were identified, five of which assessed hospitalisations as an outcome. The review reported that lower gait speed was associated with higher likelihood of secondary care utilisation.

## Discussion

### Summary

This review identified that the majority of evidence for the effect of frailty assessment in primary health care on unplanned secondary care is observational, with eight of the nine observational studies included suggesting a positive association between the identification of frailty and secondary care utilisation. Of the trial evidence, one RCT presented reduced admission rates following a health consultation programme in the home and one nRCT was ineffective in moderating hospital admissions. The reduced secondary care admission rates in the RCT suggest that its interventional care package, which incorporated frailty assessments in addition to usual care, is effective.^15^ In contrast, a multicomponent, multidisciplinary programme was ineffective at reducing admissions.^14^

### Strengths and limitations

To the best of the authors’ knowledge, this is the first review to explore frailty assessment in primary health care and its association with unplanned secondary care use. However, due to the lack of related research including secondary care use as an outcome, this focused review yielded a limited number of eligible studies. Additionally, due to time constraints, this rapid review searched only Medline and Medline In-Process, which may have restricted the number of eligible studies identified.

The three trials included in this review had methodological issues that reflect the limited quality of evidence in this area.

### Comparison with existing literature

The latest best practice guideline by the BGS, recommends the following tools for triaging individuals for a CGA: gait speed and the timed-up-and-go test for clinical staff during routine assessment; and the PRISMA-7 questionnaire for self-assessment tests.^1^ However, this recommendation is based on a review exploring test characteristics of tools for identifying frailty^23^ but which did not explore association with hospital admission.

A 2006 systematic review^24^ explored the characteristics of published CGA interventions across a variety of settings and the association with emergency department utilisation, as opposed to hospital admissions. Interventions within secondary care were mostly short-term and had little effect, whereas most interventions in primary care, outpatient, and home-care settings reduced emergency department utilisation. Like in the current review, there was heterogeneity among the included interventions; this precluded the use of meta-analysis.

### Implications for research

Perhaps unsurprisingly, this review suggests that older people identified as frail in a primary healthcare setting are more likely to be admitted to hospital, based on the observational evidence. However, from the small number of trials identified, it is not possible to draw firm conclusions on the relationship between frailty assessment and subsequent management and prevention of hospital admissions in frail older individuals, nor to recommend a specific tool. The recent introduction of the eFI to GP clinical systems in the UK may prove useful in automatically identifying more at-risk patients during routine encounters. However, how the wider primary healthcare community receive the eFI and how it can translate into effective frailty management strategies is yet to be ascertained, due to the limited time since its introduction.

An important consideration for frailty assessment in primary health care is not only its validity, but the feasibility and practicality of application. The frailty assessments described in the literature varied in mode of delivery and application, offering a variety of approaches for consideration by primary health care. However, there was heterogeneity with regard to the frailty tools utilised in the observational evidence; for instance, the CHS criteria and the EASY-Care TOS were the only tools utilised more than once in the nine studies. Additionally, where predictive validity was reported, it was generally low (reported as ‘poor’^12,19^ or ‘fair’^2^).

Although encouraging, evidence for the effect of primary healthcare frailty assessments on unplanned hospital admissions is limited and brings to question whether investment by commissioners is warranted. However, both the frail older population and associated unplanned hospital admission rates are continuing to increase. More robust research is needed on how to address frailty in primary health care as well as the acceptability of assessment tools to the primary healthcare workforce.
